# Glucose Transporter Type 1 Deficiency Syndrome: Clinical Variability and Challenges in Ketogenic Diet Management in a Paediatric Case Series

**DOI:** 10.3390/jcm15166419

**Published:** 2026-08-19

**Authors:** Mariana Viegas, Nanci Baptista, Fátima Martins, Joana Almeida, Cristina Pereira, Filipa Rodrigues, Joana Salgado, Joana Ribeiro, Sara Ferreira, Rui Diogo

**Affiliations:** 1 Inherited Metabolic Diseases Reference Centre, Coimbra Local Health Unit, MetabERN, 3004-561 Coimbra, Portugal; nancibaptista@ulscoimbra.min-saude.pt (N.B.); fatima.martins@ulscoimbra.min-saude.pt (F.M.); joanaalmeida@ulscoimbra.min-saude.pt (J.A.); saralopesferreira@ulscoimbra.min-saude.pt (S.F.); 11516@ulscoimbra.min-saude.pt (R.D.); 2 Oeste Local Health Unit, Pediatric Department, 2500-176 Caldas da Rainha, Portugal; 3 Coimbra Local Health Unit, Department of Paediatric Neurology, Paediatric Hospital of Coimbra, 3000-602 Coimbra, Portugal; 9321@ulscoimbra.min-saude.pt (C.P.); 10675@ulscoimbra.min-saude.pt (J.R.); 4 Refractory Epilepsy Reference Centre, Coimbra Local Health Unit, 3004-561 Coimbra, Portugal; 5 Faculty of Medicine, University of Coimbra, 3000-548 Coimbra, Portugal; 6 Neurodevelopment Unit, Aveiro Local Health Unit, Department of Paediatrics, 3814-501 Aveiro, Portugal; filipa.a.rodrigues@gmail.com; 7 Coimbra Local Health Unit, Department of Medical Genetics, Paediatric Hospital of Coimbra, 3000-602 Coimbra, Portugal; jrosmaninho.salgado@ulscoimbra.min-saude.pt

**Keywords:** epilepsy, glucose transporter type 1 deficiency syndrome, ketogenic diet, movement disorders, SLC2A1

## Abstract

**Background/Objectives**: Glucose transporter type 1 deficiency syndrome (GLUT1-DS) is a rare neurometabolic disorder caused by pathogenic variants in the SLC2A1 and a treatable cause of epilepsy and movement disorders. We aimed to describe clinical variability, diagnostic pathways and treatment outcomes in a paediatric case series, with a focus on ketogenic diet management. **Methods**: We retrospectively reviewed five paediatric patients with genetically confirmed glucose transporter type 1 deficiency syndrome followed at a tertiary center. Clinical presentation, biochemical findings, genetic results, treatment, dietary adherence and outcomes were analyzed. **Results**: Patients presented with development delay (4/5), movement disorders (4/5) and seizures (2/5), with symptom onset between 4 and 14 months. Diagnosis occurred between 22 and 54 months. Pathogenic SLC2A1 variants were identified in all patients, de novo in three cases. Hypoglycorrhachia was documented in two patients. All patients were treated with a classical ketogenic diet (ratio 1:1 to 3:1). Clinical improvement was observed in four patients, including better seizure control and motor function, although ketone levels were frequently suboptimal. Treatment duration ranged from two to 17 years. Long-term adherence and dietary implementation represented major challenges. **Conclusions**: Glucose transporter type 1 deficiency syndrome shows marked clinical heterogeneity, which may delay diagnosis. The ketogenic diet is effective in improving seizure control and neurological outcomes, although long-term adherence remains a major challenge. This case series highlights practical difficulties in diagnosis and dietary management in real-world clinical settings.

## 1. Introduction

The human brain relies on glucose as its primary energy source, accounting for about 20% of total body energy use [1,2]. Glucose transport across the blood–brain barrier (BBB) is primarily mediated by glucose transporter type 1 (GLUT1), a high-affinity transporter encoded by the SLC2A1 gene [2,3]. GLUT1 Deficiency Syndrome (GLUT1-DS, OMIM #606777) is a rare neurometabolic disorder caused by impaired glucose transport across the BBB, with chronic neuroglycopenia and disrupted brain metabolism [3,4,5]. SLC2A1 variants are identified in most cases (~70–90%), typically occurring de novo or inherited in an autosomal dominant manner, leading to a state of haploinsufficiency [6].

GLUT1-DS was first described in 1991 by De Vivo et al., who reported two children with early-onset drug-resistant seizures, acquired microcephaly, global developmental delay, movement disorders and low cerebrospinal fluid (CSF) glucose levels with a low CSF-to-plasma glucose ratio [7].

GLUT1-DS presents with a broad phenotypic spectrum, from mild epilepsy or isolated movement disorders to severe developmental and epileptic encephalopathy [8,9]. The classical phenotype includes early-onset, drug-resistant seizures, cognitive impairment with acquired microcephaly, and movement disorders such as ataxia, dystonia, and dyskinesias, which become the main feature in adolescents and adults [8,9,10]. Seizures typically begin before the age of two, most often as generalized epilepsy, though focal seizures and epileptic spasms may also occur [8,9]. Early-onset absence or myoclonic–atonic seizures may indicate underlying SLC2A1 variants [8,9,10,11]. Non-classical phenotypes include paroxysmal or persistent movement disorders, such as paroxysmal exercise-induced dyskinesia, chorea, and intention tremor; dysarthria, dysmetria, writer’s cramp, and transient paralysis [10,11,12], as well as hemiplegic migraine, alternating hemiplegia of childhood, and stroke-like episodes [13,14].

The ketogenic diet (KD) is an effective treatment, providing ketone bodies as an alternative cerebral energy substrate [3,8].

## 2. Methods

We conducted a retrospective descriptive case series of five consecutive paediatric patients with genetically confirmed GLUT1 deficiency syndrome (GLUT1-DS) followed at the Inherited Metabolic Diseases Reference Centre, Coimbra Hospital and University Centre, Portugal, between 2008 and 2026. The aim of this study was to describe the clinical presentation, genetic findings, ketogenic dietary management, and long-term outcomes of these patients, while highlighting practical challenges in diagnosis and treatment. Clinical, biochemical, neuroimaging, genetic, and treatment data were retrospectively collected from the medical records. Clinical improvement was assessed based on documentation in the medical records and included changes in seizure frequency, movement disorders, developmental progress, functional abilities, and caregiver-reported clinical status following initiation of ketogenic dietary therapy. The classical ketogenic diet was used as the initial dietary therapy in all patients, reflecting the standard institutional protocol during the study period. Ketogenic ratios were subsequently individualized according to age, clinical response, nutritional status, tolerance, and long-term adherence.

Blood beta-hydroxybutyrate (BHB) concentrations were measured using capillary blood samples and recorded by caregivers at home to monitor dietary adherence. BHB levels were also assessed at every outpatient visit and used to guide dietary adjustments. Follow-up visits were scheduled monthly during the initiation of ketogenic dietary therapy and subsequently every 3–6 months, according to clinical stability and individual patient needs.

In all cases, the diagnosis was established based on the clinical phenotype and identification of pathogenic SLC2A1 variants. Sequence variants are described according to Human Genome Variation Society (HGVS) nomenclature. Variant interpretation followed the American College of Medical Genetics and Genomics/Association for Molecular Pathology (ACMG/AMP) guidelines.

Neurodevelopment was assessed in all patients using the Griffiths Mental Development Scales and, in four patients, the Vineland Adaptive Behavior Scales. Developmental delay was considered present when documented in the medical records based on standardized developmental assessment and/or specialist neurodevelopmental evaluation at presentation.

Written informed consent was obtained from the parents or legal guardians of all patients. This manuscript was prepared in accordance with the CARE reporting guidelines, where applicable.

## 3. Results

### 3.1. Patient 1

A 15-month-old female was referred for evaluation of global developmental delay, mild axial hypotonia, and suspicion of epilepsy based on two apyretic generalized tonic–clonic (GTC) seizures at 13 months of age. The electroencephalogram (EEG) was normal. There was no relevant personal or family history. She was exclusively fed orally, with no history of enteral tube feeding.

She was admitted at 16 months after four GTC seizures. Two further episodes led to initiation of valproic acid (VPA) treatment, achieving seizure control. Repeated EEGs were normal, and VPA was maintained from 1.5 to 3.5 years old.

She presented with axial hypotonia and a spastic, ataxic gait. Her head circumference decreased from the 50–85th to the 3rd–15th percentile. Whole-exome sequencing (WES) revealed a pathogenic variant in the SLC2A1 gene (Table 1). She began treatment with the classical KD, with significant improvements in her motor and language skills (Table 2). The KD was initiated at a 1:1 ratio and progressively increased to 3:1 according to tolerance, ketone levels, and clinical response. Despite dietary adjustments, blood beta-hydroxybutyrate (BHB) levels were variable and frequently remained below the expected therapeutic range (maximum BHB 3.5 mmol/L, median 1.1 mmol/L), partly due to difficulties with adherence and acceptance of ketogenic supplements.

At 3.5 years, her developmental quotient (DQ) was 48. The fifth EEG, at the age of five, following an episode of oromandibular dyskinesia, revealed generalised paroxysmal activity (PA), consistent with a developmental and epileptic encephalopathy. At her last visit, aged 5.5 years, after 18 months on KD, she had achieved seizure control with some neurodevelopmental improvement, although fluctuating gait ataxia persisted. She remained on an approximately 2.7:1 KD regimen with ongoing dietary supervision.

### 3.2. Patient 2

A 15-month-old male was referred due to paroxysmal eye–head movements that had been present since four months of age. A normal EEG was recorded on two occasions. His personal and family history was unremarkable. Clinical examination and brain magnetic resonance imaging (MRI) were normal. He was fully orally fed, without the need for enteral nutritional support.

The patient developed myoclonic jerks at two years and three months of age. EEG revealed generalised PA, with segmental myoclonic seizures. Treatment with levetiracetam (LVT) led to clinical improvement. At age 3, cognitive assessment revealed a DQ of 106.

**Figure 1:**
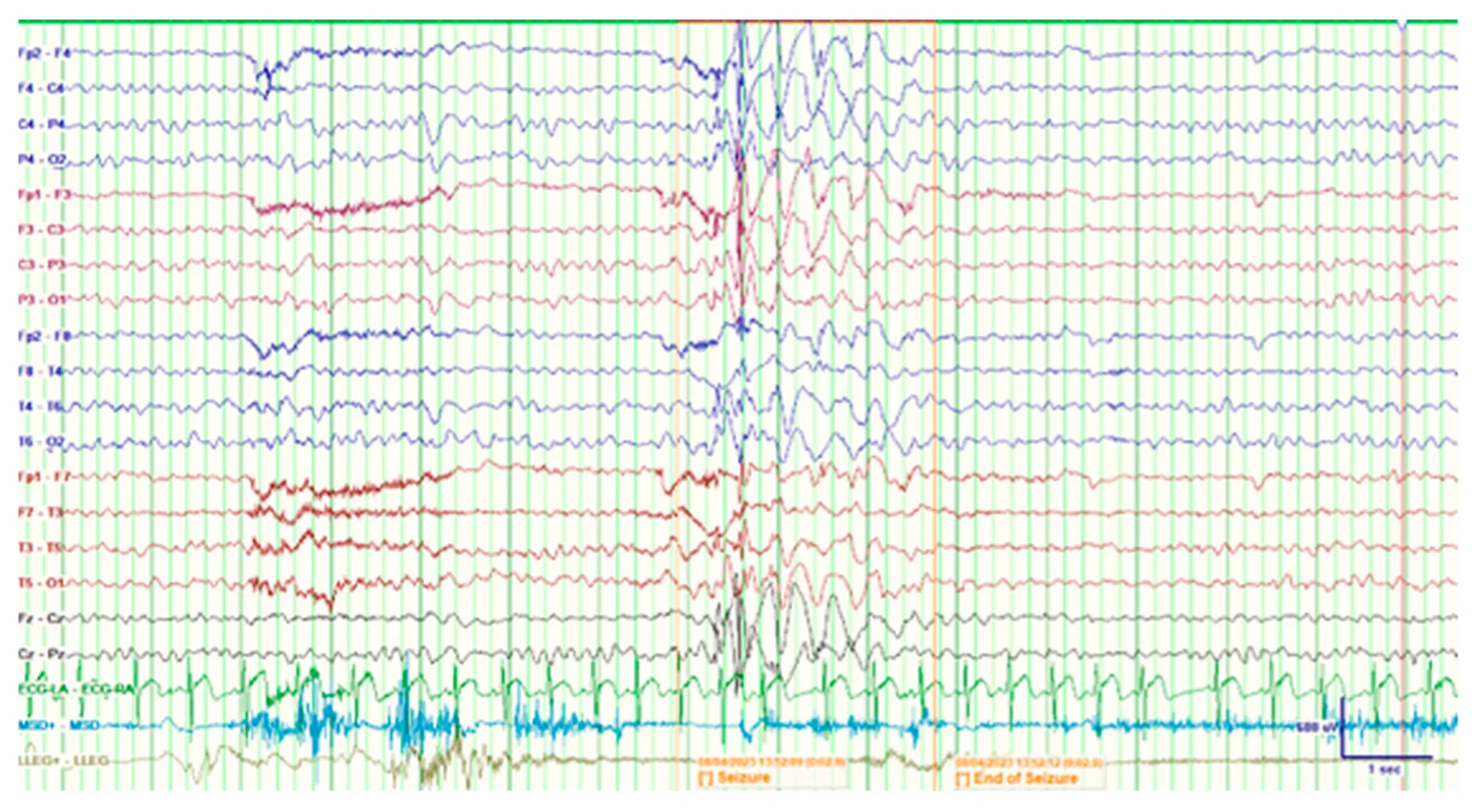
Absence seizure during hyperventilation with behavioural arrest, fixed gaze and oral automatisms. Ictal electroencephalogram showing generalized 3 Hz spike-and-wave discharges lasting 3 s.

From the age of 3.5 years, he began to experience absence seizures, as evidenced by generalised 3–4 Hz spike-wave discharges on his EEG (Figure 1). Meanwhile, dyskinesia and coordination difficulties became apparent. VPA and ethosuximide (ETX) were added sequentially to the LVT treatment. An epilepsy panel based on next-generation sequencing (NGS) identified a pathogenic variant in the SLC2A1 gene (Table 1). He started classic KD with good adherence, with a reduction in seizure frequency and a notable improvement in the motor domain (Table 2). The KD was initiated at a 1:1 ratio and progressively increased to 2.5:1 over the following months to optimize ketosis and seizure control. Initial BHB levels were low (<1 mmol/L), prompting gradual dietary adjustments. Subsequent BHB values generally ranged between 0.8 and 3.2 mmol/L, with parallel improvement in absence seizures, balance, behavior, and dyskinetic episodes.

At the age of five years and three months, his DQ was 88. The EEG showed focal centrotemporal and generalised PA. By his last visit, aged five years and nine months, he had been on the KD for 18 months. He was taking ETX and VPA and had achieved seizure control, with balance improvement and no longer experiencing dyskinetic episodes. Due to the favorable clinical response, ASM reduction is currently being considered.

### 3.3. Patient 3

A 22-month-old female patient was referred for evaluation of global developmental delay and language regression with unremarkable gestational and perinatal history. Her mother had recently been diagnosed with GLUT1-DS after a longstanding history of fluctuating ataxia, dysmetria and dysarthria, exacerbated by fasting and physical exertion. She had an autism spectrum disorder (ASD), with poor eye contact, a lack of communicative intent and guttural vocalisations. Despite having walked independently for two months, her gait was still severely ataxic. Her DQ was 43. She maintained oral feeding throughout follow-up, with no requirement for enteral feeding. The EEG was normal. Following performance of an LP, with hypoglycorrhachia, she was promptly started on a classical KD, to which she adhered well. The KD ratio was progressively adjusted according to tolerance and clinical response, eventually reaching 1.75:1, with satisfactory adherence and clinical improvement in motor and behavioral domains. The familial presentation prompted genetic investigation of both mother and daughter. SLC2A1 gene study revealed the same pathogenic variant as her mother (Table 1), confirming autosomal dominant transmission. After KD initiation, there was improvement in her attention, speech, fine motor skills and gait (Table 2). At 6.5 years, her DQ was 62. At the most recent follow-up, at seven years of age, she remained on a well-tolerated 1.6:1 KD but continued to exhibit moderate gait ataxia. She subsequently developed GTC seizures, with an EEG showing generalized PA, consistent with developmental and epileptic encephalopathy. Although an increase in the ketogenic ratio was considered, it was not pursued due to the patient’s satisfactory functional status, good dietary tolerance, and concern that a more restrictive regimen could compromise long-term adherence and nutritional acceptance. LVT was therefore introduced, resulting in seizure control.

### 3.4. Patient 4

A three-year-old male patient was referred for global developmental delay, evident since he was nine months old. He also had post-natal microcephaly. His mother and maternal grandmother had a history of intellectual disability and epilepsy. The maternal grandmother and a maternal uncle also exhibited paroxysmal dyskinesia. At the age of five, his DQ was 72, with the lowest score in Practical Reasoning (DQ 54). There was no prior history of seizures, and the EEG was normal. He was fed orally and did not require enteral tube feeding at any stage. WES identified a heterozygous pathogenic variant in the SLC2A1 gene (Table 1). The same variant was found in his mother, grandmother and uncle. This multigenerational familial clustering supported autosomal dominant inheritance with variable phenotypic expression.

The patient had substantial difficulties adhering to the prescribed KD, which prevented escalation of the ketogenic ratio and maintenance of sustained ketosis. BHB levels remained persistently low, likely contributing to the limited clinical benefit observed. Family-related challenges, including cognitive impairment in affected relatives responsible for meal supervision, also negatively impacted dietary compliance. No sustained clinical improvement was observed during follow-up, in parallel with persistently low blood ketone concentrations resulting from poor dietary adherence (Table 2).

He developed paroxysmal dyskinesia at ages 8 and 11 and has been on methylphenidate for attention-deficit/hyperactivity disorder since age 9. At his most recent evaluation at 11 years of age, his Vineland Composite score was 47.

### 3.5. Patient 5

A 21-month-old male presented with intermittent ataxia, frequent falls, irritability, and paroxysmal eye-head movements since the age of 10 months, improving after feeding. Examination showed an ataxic gait. Development was normal except for mild motor delay (walking at 19 months), and head circumference declined from the 50th to the 5th percentile. EEGs were normal, and two LPs confirmed hypoglycorrhachia (Table 1).

Based on clinical suspicion of GLUT1-DS, a classical KD was initiated. Within 15 days, caregivers reported fewer falls and behavioural improvement. During follow-up, improvement in gait stability and paroxysmal symptoms was observed while nutritional ketosis was maintained. The patient was maintained on a long-term KD, with gradual adjustments of the ketogenic ratio according to clinical response and tolerance, eventually reaching 3:1.

He remained on full oral feeding, without enteral nutritional support. After nine months, gait stability had improved and head growth had normalised. Genetic testing of fibroblasts confirmed a heterozygous variant in the SLC2A1 gene. He developed intellectual disability (DQ 60 at 2 years and 9 months; Vineland Composite 24 at 16 years and 11 months). He experienced paroxysmal dyskinesia with occasional gait impairment and sporadic GTC seizures since adolescence, with consistently normal EEGs (Table 2). At 18 years, he had an ataxic gait, dysarthria, and a moderate head tremor, and remained on a 2.5:1 KD with moderate compliance, using clonazepam as needed.

## 4. Discussion

### 4.1. Clinical Manifestations

Patients with GLUT1-DS exhibit a variety of neurological manifestations, including drug-resistant epilepsy typically beginning in early childhood, developmental delay, movement disorders, and acquired microcephaly [3,8,10]. The increasing recognition of atypical presentations has expanded the known clinical spectrum [6,10,11]. This case series highlights practical challenges in diagnosis and long-term dietary management of GLUT1-DS in real-world clinical settings.

Our patients presented with clinical manifestations at an early age (four to 14 months), which has been associated with a more severe phenotype in previous studies [3,6]. At diagnosis, four patients presented with neurodevelopmental delay and movement disorders, and two with seizures. A slowdown in head circumference growth was observed in three patients. Patient 3 presented with ASD, a feature previously reported in association with GLUT1-DS [6,11].

Movement disorders were observed in four patients at diagnosis. Patients 2 and 5 exhibited abnormal eye-head movements. It can be challenging to classify motor disturbances in young children, particularly in the presence of neurodevelopmental delay. The movement disorder resolved under KD in Patient 2 but evolved into paroxysmal dyskinesia in Patient 5.

In our cohort, only two patients presented with seizures. These findings contrast with the classical presentation of GLUT1-DS in early infancy, which is typically dominated by drug-resistant seizures [3,10].

As reported by Leen et al., two main phenotypes have been described: a classical form, predominantly characterized by epilepsy, and a non-classical form, mainly presenting with developmental delay and movement disorders in the absence of seizures [11,15]. This atypical phenotype was first described by Overweg-Plandsoen et al. in a 6-year-old boy with developmental delay, dysarthria, ataxia, and dystonic posturing of the arms, without seizures [15]. At that time, GLUT1-DS was predominantly recognized as an epileptic disorder.

Throughout the progression of the disease, all patients in our cohort, except Patient 4, experienced clinical seizures. It can be challenging to differentiate epileptic seizures from dyskinetic episodes, particularly in infants [10,11]. Patient 5 experienced sporadic GTC seizures despite normal interictal EEG. Patient 2 presented with paroxysmal eye-head movements and normal neurodevelopment, later developing refractory myoclonic and absence seizures before the age of four. This is consistent with reports that most patients with GLUT1-DS present with multiple, drug-resistant seizures, particularly early-onset absence seizures [8,9]. While the interictal EEG is often normal, age-dependent patterns may emerge, including slowing and focal discharges in infants and generalized 2.5–3.5 Hz spike–wave activity in children over two years of age [9], as observed in this case (Figure 1).

Byrne et al. described cases initially with normal development [16], in contrast to the more severe early presentations historically associated with epileptic encephalopathy [3,10]. Patient 1 experienced GTC seizures and achieved seizure control with VPA, and while the seizure phenotype was atypical, the presence of global developmental delay, microcephaly, and ataxia supported a diagnosis of GLUT1-DS.

Paroxysmal eye-head movements were a notable early feature in our cohort, as observed in Patients 2 and 5. These abnormal oculomotor episodes are among the most characteristic early manifestations of GLUT1-DS and often improve with age [8]. In Patient 2, these episodes occurred at four months of age, nearly two years prior to seizure onset. Pearson et al. reported a 32% prevalence of paroxysmal eye-head movements in GLUT1-DS, with 83% occurring before six months of age and 63% preceding seizure onset [17]. These findings likely reflect the early metabolic vulnerability of the developing visual system, highlighting the importance of early recognition and treatment during critical neurodevelopmental stages.

Another GLUT1-DS hallmark is decelerated head growth or postnatal microcephaly, reflecting impaired brain development during early life [3,6,10]. Earlier studies have reported a prevalence of microcephaly of approximately 50% in patients with GLUT1-DS [10,11]. However, recent data by Van Gemert et al. reported a significantly lower prevalence of overt microcephaly. Nevertheless, 75.9% of patients had head circumferences below the population mean [18], suggesting that individuals with GLUT1-DS tend to have reduced head growth compared to healthy peers. In our cohort, a slowdown in head circumference growth was observed in three patients, with microcephaly in one (below the 3rd percentile).

### 4.2. Diagnosis

The diagnosis of GLUT1-DS is based on clinical features, hypoglycorrhachia, and identification of a heterozygous pathogenic SLC2A1 variant [3]. In some cases, clinical and biochemical findings, particularly with a favorable response to KD, may support diagnosis before genetic confirmation [3,6]. CSF analysis remains essential, as hypoglycorrhachia provides direct evidence of impaired glucose transport, especially when genetic results are inconclusive, such as in noncoding or regulatory SLC2A1 variants [6]. Delays in molecular testing may also postpone KD initiation [3,6].

In our cohort, although KD was initiated less than one month after diagnosis, the average delay between symptom onset and diet initiation was 3 years and 7 months (Table 1). Only Patients 3 and 5 underwent an LP, and both initiated KD after demonstration of hypoglycorrhachia and a low CSF-to-blood glucose ratio. In Patient 5, the oldest patient, KD was initiated long before the genetic diagnosis was established in fibroblasts abroad.

Currently, genetic testing is widely available, and diagnosis is often established through WES [6]. Nevertheless, early recognition of the variable phenotypes of GLUT1-DS and demonstration of hypoglycorrhachia may allow earlier initiation of KD, potentially improving clinical outcomes [3,6].

Early recognition of GLUT1-DS remains challenging because the initial manifestations are often subtle, non-specific, and evolve over time. General paediatricians should suspect GLUT1-DS not only in infants with drug-resistant epilepsy, but also in children presenting with developmental delay, acquired microcephaly, paroxysmal eye-head movements, intermittent ataxia or dyskinesia, particularly when symptoms fluctuate with fasting, prolonged exercise, or delayed meals and improve after food intake [3,6,8,17]. Symptom fluctuation associated with fasting, prolonged exercise, or delayed meals should therefore be actively sought during history taking, as this represents an important and frequently overlooked diagnostic clue for GLUT1-DS [3,6,8,17]. Recognition of these clinical clues should prompt early referral to a paediatric neurology or inherited metabolic disease centre, where cerebrospinal fluid analysis and molecular testing can be performed promptly, allowing earlier initiation of ketogenic dietary therapy and potentially improving long-term neurological outcomes [3,6,8].

In Portugal, children with suspected neurometabolic disorders are typically first evaluated by general paediatricians or community paediatricians and subsequently referred to tertiary paediatric neurology or inherited metabolic disease centres when clinical suspicion arises. As GLUT1-DS remains a rare disorder with a broad and evolving phenotypic spectrum, early recognition may be challenging, particularly in children presenting without the classical epileptic phenotype. Increasing awareness of early clinical clues among frontline paediatricians may facilitate earlier referral, timely cerebrospinal fluid analysis and molecular testing, ultimately allowing earlier initiation of ketogenic dietary therapy. Educational initiatives aimed at increasing awareness of these early clinical features among general paediatricians may help reduce the diagnostic delay that still characterizes many patients with GLUT1-DS, including those in our cohort.

**Figure 2:**
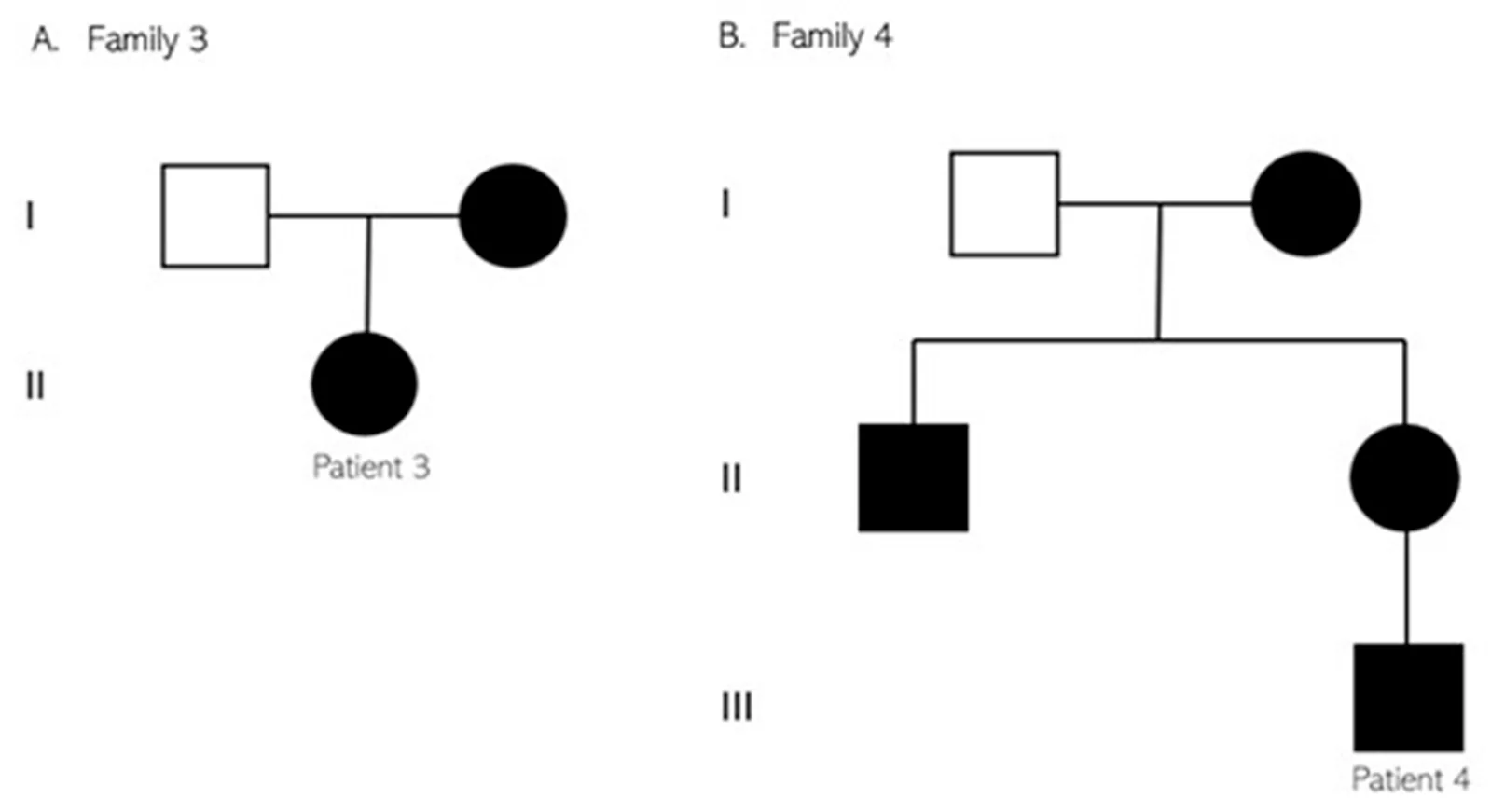
Pedigrees of Families 3 and 4 demonstrating autosomal dominant transmission of *SLC2A1*-related GLUT1 deficiency syndrome. Roman numerals (I–III) indicate generations; squares represent males and circles represent females. Filled symbols indicate affected individuals. (**A**) shows the affected mother and daughter (Patient 3). (**B**) demonstrates multigenerational transmission with marked intrafamilial phenotypic variability, including epilepsy, intellectual disability, and paroxysmal dyskinesia.

The genetic basis of GLUT1-DS is well established. In our cohort, all five patients carried a heterozygous pathogenic SLC2A1 variant (Table 1). The variants identified in Patients 2 and 4 have been previously reported [11,19]. Notably, the p.Arg333Gln variant identified in Patient 4 affects a highly conserved arginine residue involved in stabilizing intramolecular salt-bridge interactions within GLUT1, a mechanism previously associated with impaired transporter function [20]. Patient 1 carried a pathogenic nonsense variant (c.988C>T, p.Arg330*), while Patient 3 harbored a start codon variant (c.2T>C (p.(Met1?), predicted to impair protein translation, consistent with the disease mechanism [21]. In contrast to the predominantly de novo occurrence of SLC2A1 variants reported in GLUT1-DS [3,6,21], as seen in Patients 1,2 and 5, Patients 3 and 4 demonstrated autosomal dominant familial transmission, with affected relatives identified across multiple generations. Familial GLUT1-DS remains relatively uncommon and may be underrecognized, particularly in mildly affected adults presenting predominantly with movement disorders, dysarthria, or cognitive impairment rather than epilepsy [3,6]. In both families, identification of the paediatric index case prompted recognition of previously undiagnosed affected adult relatives, highlighting the marked intrafamilial phenotypic variability associated with SLC2A1 variants [22]. The pedigrees of Families 3 and 4 are shown in Figure 2.

In Patient 3, the same variant was identified in the mother, who had a history of movement disorder and dysarthria, illustrating GLUT1-DS marked intrafamilial phenotypic variability [22]. Patient 5 underwent genetic testing on fibroblasts, which identified a heterozygous SLC2A1 variant not previously reported in association with GLUT1-DS. Expert consultation highlighted that the reduced CSF glucose levels documented in this patient on two LPs provide strong functional evidence of impaired glucose transport across the BBB. The frameshift variant identified in Patient 5 was originally reported in 2008. Based on current ACMG/AMP criteria (PVS1, PS3, PM2 and PP4), the variant fulfils criteria for pathogenic classification. Although most cases of GLUT1-DS are explained by autosomal dominant SLC2A1 variants, a minority of patients may lack a confirmed genetic diagnosis despite a compatible clinical and biochemical phenotype [3,6].

### 4.3. Treatment and Outcome

GLUT1-DS is a treatable neurometabolic disorder [3]. The KD is the cornerstone of treatment, providing ketone bodies as an alternative cerebral energy source [3,8,23,24]. Early initiation is associated with improved outcomes, particularly during critical periods of brain development, although timing should be guided by clinical presentation and diagnostic confirmation [3,23,25]. However, diagnosis is often delayed, and treatment is typically initiated after symptom onset [3,8,25].

The optimal type of KD and its ketogenic ratio remain undefined and may vary according to age and individual response, with variable clinical benefit among patients [26]. Target levels of ketosis are also not well established [3,8,27]. Although generally effective, KD may affect long-term adherence due to its restrictive nature and can be associated with adverse effects such as impaired growth, nephrolithiasis, and fractures, requiring careful monitoring [26,28]. Treatment duration and management should be individualized, with some patients remaining on long-term therapy and others transitioning to less restrictive regimens [26]. Nevertheless, international consensus recommendations advocate continuation of dietary therapy into adulthood, as the benefits of ketogenic treatment in GLUT1-DS are not considered to have a defined “end-date” [3].

Resistance to antiseizure medications (ASM) is common in GLUT1-DS. Some ASMs, particularly VPA and phenobarbital, may inhibit GLUT1 function and are considered less suitable [21,29]. The KD is effective in seizure control, especially when initiated early, and may allow ASM reduction or withdrawal [3,8,9,23]. In our cohort, Patient 1 achieved seizure control with VPA prior to diagnosis, despite its reported inhibitory effect on GLUT1 in vitro [29]. Patient 2 achieved seizure control with a combination of ETX, VPA, and KD, with planned VPA withdrawal.

The impact of KD on motor and cognitive symptoms is heterogeneous and not yet fully understood [23,24,26]. Previous studies have consistently demonstrated improvement in movement disorders, including paroxysmal dyskinesia, ataxia and dystonia, following ketogenic dietary therapy in patients with GLUT1-DS [23,24,26]. Similar benefits were observed in the patients from our cohort who presented with motor manifestations and achieved sustained nutritional ketosis. In contrast, Patient 4 did not achieve nutritional ketosis (median BHB 0.1 mmol/L; maximum 0.5 mmol/L) because of marked difficulties with dietary adherence. Therefore, the absence of clinical benefit in this patient should be interpreted as unsuccessful implementation of KD therapy rather than lack of treatment efficacy. Early KD initiation, particularly in patients with preserved cognition, has been associated with improved psychomotor outcomes [23,26], as observed in Patient 2. Conversely, patients with established cognitive impairment may experience more limited benefits, with mild improvements in areas such as attention, speech, and overall cognitive function [24,26]. In our cohort, Patients 1 and 3, who both had significant cognitive impairment, showed some improvement in attention and expressive language within three months of starting KD. However, consistent with international experience in GLUT1-DS, ketogenic dietary therapies appear to have a greater impact on seizure control and movement disorders than on reversal of established cognitive impairment, particularly when treatment is initiated after symptom onset [3,26] (Table 2). All patients received educational support and adjunctive therapies according to their individual needs.

Adhering to a restrictive diet throughout life is challenging and requires substantial commitment from both families and healthcare professionals. By the time of diagnosis, many children have already established feeding behaviors and may present neurodevelopmental difficulties, selective eating habits, or behavioral issues that negatively affect adherence. Recent real-world studies highlight that flexibility is frequently required in the nutritional management of GLUT1-DS, with clinicians commonly individualizing ketogenic therapies through gradual ketogenic ratio adjustments, incorporation of medium-chain triglyceride (MCT) supplementation, adaptation of meal composition to patient preferences, use of ketogenic formulas and ketogenic-friendly recipes, and transition to less restrictive regimens when appropriate [30]. In some patients, maintaining a sustainable dietary regimen with moderate ketosis may be preferable to pursuing stricter dietary targets associated with poor adherence and reduced quality of life. Therefore, successful long-term management of GLUT1-DS depends not only on achieving ketosis, but also on maintaining a feasible and individualized dietary strategy through close multidisciplinary follow-up and continuous family support. Family support should begin at the time of diagnosis and continue throughout follow-up, as successful ketogenic dietary therapy depends heavily on caregiver engagement and education. At treatment initiation, families should receive practical counselling regarding meal preparation, ketogenic ratio calculation, food selection, ketone monitoring, recognition of potential adverse effects, and management during intercurrent illness. Regular follow-up by a multidisciplinary team, including paediatric neurologists, metabolic physicians and experienced dietitians, allows dietary adjustments according to growth, nutritional status, ketosis, and clinical response, while addressing feeding difficulties, treatment fatigue and psychosocial challenges that may emerge over time [3,26,30]. This approach is particularly important in young children and in families with additional caregiving challenges, such as cognitive impairment or neurodevelopmental disorders, as illustrated by the limited adherence observed in Patient 4 of our cohort. This distinction is clinically important, as poor adherence may prevent achievement of nutritional ketosis and consequently preclude an adequate therapeutic trial of ketogenic dietary therapy.

From a paediatric perspective, the management of GLUT1-DS should adopt a family-centred approach, recognising that long-term treatment affects not only the child but the entire household. Caregivers play a central role in maintaining dietary therapy and require ongoing education, emotional support, and shared decision-making throughout the disease course. Coordination between tertiary metabolic centres, primary care paediatricians, schools, and rehabilitation services may facilitate adherence, optimise neurodevelopmental outcomes, and improve quality of life. In addition, regular reassessment of family needs is essential, as the challenges associated with ketogenic dietary therapy evolve with the child’s age, developmental stage, and increasing social participation [3,26,30]. This approach may be particularly important when caregivers themselves are affected by GLUT1-DS or other neurodevelopmental conditions, as illustrated by one of the families in our cohort, where caregiver limitations substantially affected long-term dietary adherence.

Despite the use of the classical KD, several patients in our cohort exhibited persistently low or fluctuating BHB levels, mainly related to difficulties with dietary adherence, selective eating behaviors, or poor acceptance of ketogenic supplements. This was particularly evident in Patients 1 and 4 and may have limited the overall therapeutic benefit. Conversely, Patient 2 achieved more sustained ketosis, generally with BHB levels between 1.5 and 3 mmol/L, which paralleled improved seizure control and motor outcomes. These findings highlight the importance of individualized dietary adjustments and close nutritional follow-up in optimizing long-term KD efficacy in GLUT1-DS. Patient 4, who was placed on the KD at 4 years and 6 months of age, had a significant developmental delay and did not adhere to the diet. His mother and grandmother, both with GLUT1-DS, found it difficult to cope with the diet.

All patients underwent an extended newborn screening, including for fatty-acid oxidation disorders, which enabled the diet to be started within one month of diagnosis. Any other contraindications were ruled out, and nutritional deficiencies were monitored and addressed. As patients receiving long-term KD may develop secondary hypocarnitinaemia, regular biochemical monitoring is recommended and supplementation should be provided when indicated [3]. In our cohort, no significant hypocarnitinaemia was observed. Long-term safety monitoring included growth, lipid profile, liver function, renal complications, bone health, and biochemical nutritional markers. No patient developed nephrolithiasis or clinically significant dyslipidaemia during follow-up. Likewise, no clinically relevant impairment of growth or bone health was observed during follow-up.

Overall, our findings are consistent with current international recommendations and previous cohort studies supporting ketogenic dietary therapy as the standard treatment for GLUT1-DS [3,23,26]. In our series, KD was associated with improved seizure control and motor manifestations in most patients, including ataxia, dyskinesia and abnormal eye-head movements, mirroring previously reported outcomes [23,24,26]. Conversely, cognitive improvement was more modest, particularly in patients with established neurodevelopmental impairment before treatment initiation, reinforcing the importance of early diagnosis and timely implementation of dietary therapy during critical periods of brain development [3,23,26]. These findings support an individualized approach to ketogenic therapy, balancing the degree of ketosis with long-term adherence and quality of life rather than pursuing strict ketogenic targets in every patient [26,30]. In Patient 5, sustained nutritional ketosis was accompanied by long-term improvement in gait stability and paroxysmal symptoms, consistent with the established therapeutic effects of ketogenic dietary therapy on movement disorders in GLUT1-DS reported in previous studies [23,24,26]. Although causal inference cannot be established in a retrospective case series, this observation is consistent with previous evidence highlighting the importance of achieving and maintaining adequate nutritional ketosis to optimize clinical response.

Our findings support considering GLUT1-DS in patients presenting with early-onset epilepsy and movement disorders, particularly when accompanied by cognitive impairment, as well as in atypical phenotypes without seizures. The cerebral metabolic rate for glucose is physiologically low during fetal development and increases progressively, reaching a peak in early childhood, before declining during adolescence [1]. This developmental pattern may help explain the early onset of neurological manifestations in GLUT1-DS.

Recognizing both classical and atypical phenotypes at an early stage is essential, as the KD remains a highly effective therapy for seizure control and movement disorders in GLUT1-DS and may also improve gait stability, abnormal eye movements, attention, and behavioral symptoms [3,8,23]. However, cognitive outcomes appear to be more variable, particularly when treatment is initiated after neurodevelopmental impairment has already become established. In addition, advances in NGS, including WES, have increasingly facilitated the diagnosis of GLUT1-DS, as illustrated in our cohort, where genetic testing played a key role in identifying two affected patients.

Beyond confirming the established benefits of ketogenic dietary therapy, our series emphasizes the practical challenges of long-term implementation in routine clinical practice, particularly regarding sustained adherence, individualized dietary adjustments, and family support. Unlike previous cohorts, a relatively low proportion of patients presented with seizures at onset, reinforcing the importance of recognizing non-classical phenotypes.

This study has limitations, including the small sample size, retrospective design, and single-center setting, which may limit generalizability.

## 5. Conclusions

This retrospective case series highlights the marked clinical heterogeneity of GLUT1-DS and illustrates the importance of recognising both classical and atypical phenotypes to facilitate early diagnosis. Our experience supports the clinical benefits of ketogenic dietary therapy for seizure control and movement disorders when adequate nutritional ketosis can be achieved, while emphasizing that long-term outcomes also depend on sustained adherence, individualized dietary management and continuous family support. Although limited by its small sample size and retrospective design, this study provides practical insights into the long-term management of children with GLUT1-DS that may assist clinicians caring for these patients.

## Figures and Tables

**Table 1:** Clinical, laboratory and genetic features of patients with GLUT1-DS.

	Patient 1	Patient 2	Patient 3	Patient 4	Patient 5
Sex	F	M	F	M	M
Age at onset	14m	4m	9m	9m	10m
Age at diagnosis	3y, 11m	4y, 3m	1y, 10m	4y, 6m	2y, 8m
Seizures	Generalized tonic–clonic	Myoclonic jerks, early-onset absences	Not present	Not present	Not present
Neurodevelopment	Global delay 3y, 6m: DQ 48 (*)	Normal 3y: DQ 106 (*)	Global delay + ASD 1y, 10m: DQ 43 (*)	Global delay 3y, 6m: DQ 72 (*)	Mild motor delay (gait at 19m)
Movement Disorders	Spastic ataxia	Paroxysmal eye movements, dyskinesia, coordination disorder	Ataxic gait	Not present	Paroxysmal eye movements Intermittent ataxia
Head circumference at birth (**)	50th–85th	15th–50th	15th–50th	50th	50th
Head circumference at diagnosis (**)	3rd–15th	50th	50th	<3rd	5th
Brain MRI (age)	Normal (3y, 8m)	Normal (1y, 7m)	ND	ND	Normal (2y, 8m)
Glucose CSF; CSF/plasma (#)	ND	ND	36 mg/dL; 0.43	ND	28.8 mg/dL; 0.34
*SLC2A1* gene pathogenic variant (***); inheritance	c.988C>T (p.Arg330*) heterozygous de novo	c.1199G>A (p.Arg400His) heterozygous de novo	c.2T>C (p.(Met1?)) heterozygous inherited from her mother	c.998G>A (p.Arg333Gln) heterozygous inherited from his mother	c.848insA (p.Pro284Alafs*75) heterozygous de novo (parents tested negative)

(*) Ruth Griffiths Scale; (**) percentile; (***) variants are reported according to HGVS recommendations using transcript NM_006516.3. (#) reference values Glucose: CSF—>40 mg/dL CSF/plasma—>0.45; CSF: cerebrospinal fluid; DQ—Development quotient;; F: female; M: male; m: months; MRI: magnetic resonance imaging; ND—not done y: years.

**Table 2:** Clinical evolution of patients with GLUT1-DS under ketogenic diet.

		Patient 1	Patient 2	Patient 3	Patient 4	Patient 5
Age at start of KD		3y, 11m	4y, 3m	1y, 10m	4y, 6m	2y, 8m
KD duration/Maximum KR		1y, 7m/3:1	1y, 6m/2.5:1	5y, 2m/1.75:1	5y, 2m/1:1	15y, 4m/3:1
Ketonemia (#) limits and median		0.3–3.5 (1.1)	0.8–3.2 (1.8)	0.4–5.1 (2.7)	0–0.5 (0.1)	0.2–3.7 (1.9)
Seizures before KD		Yes	Yes	No	No	No
EEG before KD		Normal	Generalized epilepsy-childhood absence epilepsy	Normal	Normal	Normal
AED before KD		No (VPA stopped)	LVT, VPA, ETX	No	No	No
**Evolution**	Age at last visit	5y6m	5y9m	7y5m	11y	18y
	Current AED	No	ETX, VPA	LVT	No	No
	Last EEG (age)	Slow basal rhythm; bilateral parieto-occipital and generalized PA (5y)	Left centrotemporal focal PA and generalized PA during sleep (5y)	Slow basal rhythm; focal right frontal and generalized PA (7y)	Normal (11y)	Normal (17y)
	Seizures	Control	Control	GTC seizures Myoclonic jerks	No	GTC seizures
	Motor disturbance	Fluctuating ataxia	Global motor improvement No dyskinesia	Moderate ataxia Weakness episode	No	Dyskinesia; intermittent weakness
	Intellectual disability (*)	Yes (moderate)	No	Yes (moderate)	Yes (moderate)	Yes (severe)

AED: antiepileptic drugs; EEG: electroencephalogram; ETX: ethosuximide; KD: ketogenic diet; KR: ketogenic ratio (grams of fat/grams of protein + carbohydrates); Ketones: # capillary blood 3-hydroxibutiric acid (mmol/L) last 6 months controls; LVT: levetiracetam; m: months; PA: paroxysmal activity; GTC: generalized tonic–clonic; VPA: sodium valproate; y: years (*) Diagnostic and Statistical Manual of Mental Disorders- Fifth Edition Text Revision (DSM-5-TR).

## Data Availability

The data supporting the findings of this study are not publicly available due to privacy and ethical restrictions related to patient confidentiality. All relevant data supporting the findings of this study are included within the article.
